# Relationship between activity limitation and health-related quality of life in school-aged children with cerebral palsy: a cross-sectional study

**DOI:** 10.1186/s12955-017-0650-8

**Published:** 2017-04-28

**Authors:** Eun-Young Park

**Affiliations:** Department of Secondary Special Education, College of Education, Jeonju University, 1200 3-ga, Hyoja-dong, Wansan-gu, Jeonju, 560-759 South Korea

**Keywords:** Cerebral palsy, Functional classification system, Health-related quality of life

## Abstract

**Background:**

Information on health-related quality of life is becoming increasingly important in children with cerebral palsy. This study investigated the relationship between activity limitation and health-related quality of life in school-aged children with cerebral palsy.

**Methods:**

Data were collected from 71 children aged 6–15 years with cerebral palsy. Activity limitations were assessed using functional classification systems, including the Korean-Gross Motor Function Classification System (K-GMFCS) and the Korean-Manual Ability Classification System (K-MACS). Health-related quality of life was assessed using the Korean version of the Childhood Health Assessment Questionnaire. Physical therapists collected the data by interviewing the parents of the subjects.

**Results:**

Both the K-GMFCS and the K-MACS were significantly positively correlated with the Childhood Health Assessment Questionnaire. The Childhood Health Assessment Questionnaire score differed significantly with respect to the functional classification systems. The differences in the ratings according to the K-GMFCS levels were significant, except those between levels I and II, levels II and III, levels III and IV, and levels IV and V. In the K-MACS, there were no significant differences between levels I and II, levels III and IV, and levels IV and V. The K-GMFCS and the K-MACS were significant predictors of health-related quality of life, demonstrating 75.5% of the variance (*p* < 0.05).

**Conclusion:**

Comprehensive information on children with cerebral palsy should be gathered to provide professionals with a better understanding of health-related quality of life.

## Background

Cerebral palsy (CP) is a permanent disability secondary to non-progressive brain anomalies arising in the fetus or during infancy that impairs posture and restricts movements. Movement disorders of CP involve sensory, perception, cognition, and communication impairments; behavior disorders; epilepsy; and secondary musculoskeletal problems [1]. CP is well known as the most common physical disability in childhood. The range of development and comorbidities of children with CP vary. These characteristics of children with CP affect the quality of life (QOL) of the family as well as the functional outcomes [2]. The severity of CP is associated with a low physical QOL [3, 4].

The QOL references the general well-being of individuals and societies and is defined by the World Health Organization (WHO) as “an individual’s perception of their position in life in the context of the culture and value systems in which they live, and in relation to their goals, expectations, standards, and concerns” [5]. Well-being is a general term for the condition of an individual or group and is a useful concept in public health because well-being integrates mental health and physical health, resulting in a more holistic and broad approach to health promotion and disease prevention. A high QOL is associated with positive outcomes, such as decreased risk of disease, illness, and injury, better immune functioning, rapid recovery, improvement of productivity at work, and increased contribution to communities.

Health-related QOL (HRQOL) is a more specific concept than the more general social and environmental issues proposed by the WHO [6, 7] and is especially relevant to conditions that are chronic and disabling, such as CP [8]. CP has a significant effect on HRQOL during childhood. Vargus-Adams [3] reported a reduced HRQOL of children with CP, particularly in the physical function domain compared with the psychosocial domain. The correlation between CP and HRQOL, especially in the physical domain, triggered studies which investigated the relationship between function and HRQOL [4]. HRQOL measurements, such as the Childhood Health Assessment Questionnaire (CHAQ), reflect mainly functional abilities in performing activities of daily living and are related with other functional measurements [9]. Higher correlations between the scales and the domains of the CHAQ indicate that lower limb function (arising and walking) or upper limb function (reaching and gripping) might be expected. The CHAQ is simple to administer and score [10].

Functional outcome is concerned with the most important aspects resulting from disabilities in terms of an individual’s level of independence in life roles. The International Classification of Functioning, Disability, and Health (ICF) suggested mainstream ideas that individuals understand health and disability in the view of the social aspects of disability [11]. The ICF urged the definition of disability in terms of activity limitation and participation restriction rather than the etiology of the condition or disease [12]. A decrease in the functional outcome, including activity limitation, according to the terminology of the ICF is one of the most frequent clinical symptoms that have significant impacts on children with CP and on the society. The ICF model helps organize available information into a meaningful map of limitations in functioning [13]. Although the ICF model proposed activity limitation and participation restriction as different factors, the meaning of these two factors was divided specifically. The ICF described activity limitation and participant restriction into one component, and information on this component was gathered by the two qualifiers of performance and capacity. The domains for the Activities and Participation component were provided in a single list that covers the range of life areas from basic learning or watching to composite areas, such as interpersonal interactions or employment. The component can be used to represent activities or participation or both [13].

The suggestion of the ICF indicates that the functional levels and abilities of children with CP have become more important [14], and the effort to reflect the views of the ICF led to the development of functional classification systems. The functional classification systems for children with CP at these levels are valuable for evaluating outcomes after interventions and determining the impact of health care needs on the society [15]. Until now, studies investigating the relationship between functional classification systems and clinical measurements are being performed. The relationships of these classification systems with other clinical measurements, including the Gross Motor Function Measure [16] and WeeFIM [17], have been investigated. However, there are insufficient data for verifying the relationship between functional classification systems and HRQOL in children with CP. A classification system is an assessment tool primarily used in interventions for children with CP for communication across various professionals. Measuring HRQOL provides valuable insight into the perception of health status and complements more objectively collected data and investigations into the relationship between functional classification systems that assess activity limitations. Further, assessing HRQOL could connect professionals and families because this system is widely used and easily applied. The present study determined the relationship between functional classification systems and HRQOL in school-aged children with CP. This study addressed three questions regarding the purpose of study. First, what is the correlation between the functional classification systems (Gross Motor Function Classification System [GMFCS] and Manual Ability Classification System [MACS]) and the HRQOL in school-aged children with CP? Second, is the HRQOL score different in accordance with the functional classification system level? Third, how much HRQOL could be explained by the functional classification systems?

## Methods

### Research design

This study employed a cross-sectional and prospective design.

### Participants

The participants were 71 children: 49 (69.0%) boys and 22 (31.0%) girls. Their mean weight was 26.4 kg (SD = 8.1), and their mean height was 125.0 cm (SD = 14.01). Their mean age (SD) was 109.77 months (SD = 30.80). The inclusion criteria were as follows: (a) participants with a clinical diagnosis of CP, (b) age range from 6 to 15 years, and (c) agreement of parents to participate. The exclusion criteria were as follows: (a) children with spina bifida, (b) children with skeletal muscular disorders, such as muscular dystrophy and myopathy, and (c) children who have underwent elective dorsal rhizotomy surgery. Whether a botulinum toxin was injected into the patients was not considered in this study. The minimum sample size for verifying a correlation was proposed in accordance with the effect sizes and statistical power. The minimum required sample size was 42 based on a significance level of p = .0.5 and a medium effect size of .50 [18]. Thirty-eight children with CP used walking aids or assistive devices, such as canes, walkers, crutches, and wheelchairs. The type of CP was spastic in 58 (81.7%) children with CP, dyskinetic/athetotic in nine (12.7%), ataxic in one (1.3%), and mixed in three (4.2%). According to the Korean-GMFCS (K-GMFCS), 16 (22.5%), 15 (21.1%), 11 (15.5%), seven (9.9%), and 22 (31.0%) children were classified under levels I–V, respectively. According to the Korean-MACS (K-MACS), four (9.2%), 36 (50.7%), 11 (15.5%), 11 (15.5%), and nine (12.7%) children were at levels I–V, respectively.

Regarding the participating parents, 11 were men (15.5%), and 60 were women (84.5%); 10 were aged ≤29 years (14.1%), 37 were aged 30–39 years (52.1%), and 24 were aged 40–49 years (33.8%). The frequency of middle school graduates was 22.5%, and that of high school graduates was 46.7%. The frequency of above university graduates was 38.8%.

### Data collection

The data were collected in elementary schools for children with physical disabilities or who received rehabilitation therapies in hospitals. Gross motor function was classified in accordance with the K-GMFCS. The GMFCS has five ordinal levels to describe the gross motor function of children based on self-initiated movements in a clinical setting [19]. The intraclass correlation coefficient is reported to be from 0.993 to 0.996 [20]. The K-MACS also contains five ordinal levels to classify how children with CP use their hands when handling objects in daily activities. The intraclass correlation coefficients of the MACS were 0.97 among therapists and 0.96 between parents and therapists [21]. The measurements were conducted by 10 therapists (six physical therapists and four occupational therapists), each with at least three years’ experience of providing therapy for children with CP.

The CHAQ was used for measuring the HRQOL of the school-aged children with CP. The Korean version of the CHAQ was assessed by physical therapists by interviewing the parents. The CHAQ consists of eight domains, including dressing/grooming, arising, eating, walking, hygiene, reaching, gripping, and activities. The questions were answered and scored on a scale of 0 to 3 (0 = able to do activities with no difficulty, 1 = able to do activities with some difficulty, 2 = able to do activities with much difficulty, and 3 = unable to do activities). The mean of the eight scores determined the CHAQ score (range 0–3). The Cronbach’s alpha ranged from 0.76 to 0.97 for the eight domains [22], and values of 0.880–0.973 were reported in children with CP [23].

### Statistical analysis

Descriptive statistics were used to examine the general characteristics of the children with CP. The Spearman rank correlations were used to analyze the correlations between the functional classification systems and HRQOL. The mean difference in participation according to the functional classification system level was analyzed using a one-way analysis of variance. A stepwise multiple regression analysis was applied to investigate the effects of the functional classification systems on participation. The level of significance was set at *p* < 0.05.

## Results

The correlations between the CHAQ score and K-GMFCS and K-MACS are presented in Table 1. All coefficients were significant (*p* < 0.01). The lowest correlation coefficient was 0.378 between the K-GMFCS and dressing/grooming, and the highest was 0.825 between the K-GMFCS and the CHAQ score.

**Table 1:** Correlations between the CHAQ score and functional classification systems

	Dressing/grooming,	Arising	Eating	Walking	Hygiene	Reaching	Gripping	Activities	CHAQ
K-GMFCS	.378^**^	.852^**^	.474^**^	.809^**^	.689^**^	.757^**^	.584^**^	.591^**^	.825^**^
K-MACS	.479^**^	.618^**^	.530^**^	.460^**^	.553^**^	.698^**^	.656^**^	.368^**^	.731^**^

*K-GMFCS* Korean-Gross Motor Function Classification System, *K-MACS* Korean-Manual Ability Classification System, *CHAQ* Childhood Health Assessment Questionnaire

^**^ *p* < 0. 01

The mean CHAQ score and each domain score according to the functional classification levels I–V are shown in Table 2.

**Table 2:** Mean CHAQ score according to the functional classification system level

	K-GMFCS					*p*
	I	II	III	IV	V	
CHAQ	10.57 ± 7.20	27.57 ± 20.58	38.36 ± 15.65	57.20 ± 18.60	72.05 ± 18.41	.000
	K-MACS					*p*
	I	II	III	IV	V	
CHAQ	17.25 ± 4.57	25.00 ± 18.63	54.60 ± 18.06	55.86 ± 33.20	82.63 ± 9.52	.000

*K-GMFCS* Korean-Gross Motor Function Classification System, *K-MACS* Korean-Manual Ability Classification System, *CHAQ* Childhood Health Assessment Questionnaire

The mean CHAQ score differed significantly in accordance with the K-GMFCS level (*p* < 0.05). The post hoc analysis showed significant differences between K-GMFCS levels I and III and levels IV and V. K-GMFCS level II showed significantly lower scores than levels IV and V. The score at K-GMFCS level III was significantly lower than that at level V. The mean CHAQ score also differed significantly in accordance with the K- MACS level (*p* < 0.05). Furthermore, the post hoc analysis showed significant differences between K- MACS levels I and III and levels IV and V. K- MACS level II showed significantly lower scores than levels III, IV, and V. The score at K- MACS level III was significantly lower than that at level V.

The regression analysis showed that K-GMFCS and K-MACS were significantly associated with the CHAQ score (*p* < 0.05). The model explained 75.5% of the variance (Table 3).

**Table 3:** Regression analysis: factors affecting the CHAQ score

Model		Unstandardized coefficients		Standardized coefficients	t	*p*	Adjusted R^2^
		B	Standard error	Beta			
CHAQ	Constant	−16.53	4.74		−3.47	.001	0.755
	K-GMFCS	11.14	1.49	0.60	7.48	.001	
	K-MACS	8.82	1.97	0.36	4.49	.009	

*K-GMFCS* Korean-Gross Motor Function Classification System, *K-MACS* Korean-Manual Ability Classification System, *CHAQ* Childhood Health Assessment Questionnaire

## Discussion

Outcome measurement tools are used to evaluate functional performance, plan treatment goals, and interpret outcomes [24]. The interest on the relationship between tools increased in accordance with the emphasis of evidence-based research [25]. Many clinicians still attempt to determine the meaning of outcome measure results and how to integrate this information into clinical practice [24]. Given the possibility of the use of HRQOL as an outcome in clinical trials, studies that would link clinical variables with HRQOL are needed [26].

Among clinical variables, functional classification systems, such as the GMFCS and MACS, have been commonly used in clinical settings. Moreover, the functional classification systems are assessment tools primarily used in the interventions for children with CP for communication across various professionals. The investigation of the relationship between the functional classification systems and HRQOL in children with CP could be helpful in enabling clinicians understand HRQOL and communicate with teachers and parents. To provide information on the relationship between activity limitation and HRQOL, we analyzed the data of the K-GMFCS, K-MACS, and CHAQ in school-aged children with CP.

Information on HRQOL might be useful to evaluate the effects of interventions on the overall well-being [27]. Because measures of HRQOL may be used to predict the future status of individuals with an illness, these could be used as a better indicator than functional outcome assessments in children with CP who attend school [28]. HRQOL is an interchangeable term and includes health status and functional status, which refers broadly to the physical functioning dimension. However, the HRQOL construct also includes the psychosocial, emotional, social, and role functioning [29]. Clinicians tend to focus more on interventions to improve the functional status, such as gross motor, fine motor, and activities of daily living and assessment of functional status. There might be significant barriers to the routine clinical use of HRQOL [29].

In previous studies, the usefulness of tools or classification systems is used to provide evidence of reliability and validity [30, 31]. The relationship with the classification system can be used to provide a basis for construct validity of instruments. What are often suggested in the review of the validity of measurement tools are grounds for other variables. The rationale based on other variables known as construct validity is to validate the validity of the test by analyzing the relationship between test scores and external variables [32]. The rationale for construct validity can be presented by examining the static correlation between the concept to be measured and the same test [33]. In this study, we looked at correlations between the functional classification systems and HRQOL, with an emphasis on examining which relationships exist among the different types of assessments and what degree. The K-GMFCS and K-MACS, which measure activity limitation, were significantly correlated with the CHAQ, which measures the HRQOL (*r* = 0.378 to 0.825). The strongest correlation was shown between the K-GMFCS and CHAQ. The K-GMFCS showed a higher correlation coefficient than the K-MACS, except for the dressing/grooming and eating domains. These results are consistent with those of previous studies. Gunel et al. [34] reported that there was a significant correlation between the GMFCS, MACS, and WeeFIM subscales. In a detailed analysis, the highest correlation scores were between the self-care subgroup of the WeeFIM and MACS and between the locomotion subset of the WeeFIM and GMFCS. HRQOL describes an individual’s perceived ability to participate in physical and social activities in their environment and their level of enjoyment or satisfaction in that involvement given their disease or health status. Functional outcomes, such as activity limitations, are a concept that is related to HRQOL but have a distinct definition [35]. The results of the correlation from medium to large between the functional classification systems and CHAQ are consistent with similar but concept of activity limitation and HRQOL. Based on the findings from the present study, moderate to strong relationships exist between the functional classification levels and scores obtained from the HRQOL, and these associations provide evidence that the assessed HRQOL can distinguish between children with different levels of CP, and clinicians should feel comfortable using HRQOL to assess the functional levels in children with CP [25].

The reason which verifies the differences according to the GMFCS level was that the GMFCS is rapidly being accepted in clinical practice and research [36] and has been reported to be directly related to the limitations of activity and participation [15]. One example was a study which examined the discriminant validity of Pediatric Evaluation Disability Inventory based on the GMFCS level. That study reported that scores decreased significantly with increasing GMFCS levels for all domains of the PEDI [37]. The mean CHAQ score differed significantly in accordance with the functional classification system level. In both the K-GMFCS and K-MACS, the CHAQ score differed significantly between levels I and III and levels IV and V. Level II showed significantly lower scores than levels III, IV, and V. The score at level III was significantly lower than that at level V. In summary, there was no difference between levels I and II and levels IV and V. The results of this study were consistent with those of previous studies which investigated the associations between QOL and functioning in children with CP. Shelly et al. [9] examined the relationship between the GMFCS and QOL and reported a significant correlation.

A previous study examined whether outcome variables could predict the GMFCS level of children with ambulatory CP by calculating scale scores according to the GMFCS level [25]. In this study, the multiple regression analysis was employed to investigate the effects of activity limitation on the HRQOL. The K-GMFCS (*β* = 0.60) and K-MACS (*β* = 0.36) explained 75.55% of the CHAQ variance. The K-GMFCS and K-MACS scores significantly affected HRQOL, and the explained variance was large. However, the remaining 24.45% of variance of the CHAQ indicates that there is a possibility that other factors affecting HRQOL in children with CP, such as environmental factors and context, exist. In considering this result, another factor which affected the HRQOL in children with CP, except activity limitation, should be verified for better understanding. Fewer measures of functional impairment have been developed compared to HRQOL, and few research studies are conducted on functional outcome assessment tools [35]. However, in children with CP, the situation of functional outcome and HRQOL was reversed. HRQOL data have many potential applications in research and clinical care. The data of the relationship between activity limitation and HRQOL could increase the applicability of the CHAQ in clinical settings.

Approach, intervention, and education for children with CP should be performed by a multidisciplinary team. Clinicians are more familiar with medically oriented terms, and other professionals and parents are unfamiliar with medical terms. The functional classification system is useful for describing terms, and functional criteria are useful for making direct comparisons with typical behaviors and activities. In addition, this system provides guidelines that would foster communication among professionals. The relationship between the functional classification system and HRQOL might promote the understanding of well-being in children with CP and provide clinicians with guidance for interpreting the HRQOL scores.

There were limitations in this study. The first limitation was the sample size. Although this study used the minimum guideline of the sample size for a correlation study, higher numbers of children with CP would increase the statistical power and clinical meaning of the results of this study. The second limitation was the age range of the children with CP. There is a possibility that the 9 years of motor development are an important contribution to activities and performance. Although the correlation between age and score of the CHAQ was not significant (*r* = .039) in the additional analysis, a further correlation analysis controlling for age should be considered in future studies. Another factor that should be considered in further studies is the type of CP. The characteristics of CP, such as decreased muscle tone, were different in accordance with the types of CP investigated in this study, which may affect the results. In this study, the effect of the type of CP was not analyzed because each type of CP was not arranged in accordance with each functional classification level owing to the small sample size. The relationship between activity limitation and HRQOL in specific types of CP should be verified in further studies.

## Conclusions

This study investigated the relationship between activity limitations and QOL, as proposed by the ICF. Data were collected in 71 school-aged children with CP. The mean differences in the CHAQ score were significant in accordance with the functional classification system levels. The K-GMFCS and K-MACS had a moderate to large correlation with the CHAQ score and were considered factors affecting the HRQOL. Although the functional classification systems, which measure activity limitations, showed significant correlations and explained a large HRQOL variance, more comprehensive information on children with CP should be gathered to provide professionals with a better understanding of HRQOL.

## Abbreviations

CHAQ: Childhood health assessment questionnaire
CP: Cerebral palsy
HRQOL: Health-related quality of life
ICF: International classification of functioning, disability, and health
K-GMFCS: Korean-gross motor function classification system
K-MACS: Korean-manual ability classification system
QOL: Quality of life
WHO: World Health Organization
